# Consensus statement from the international consensus meeting on post-traumatic cranioplasty

**DOI:** 10.1007/s00701-020-04663-5

**Published:** 2020-12-22

**Authors:** C. Iaccarino, A. Kolias, P. D. Adelson, A. M. Rubiano, E. Viaroli, A. Buki, G. Cinalli, K. Fountas, T. Khan, S. Signoretti, V. Waran, A. O. Adeleye, R. Amorim, A. Bertuccio, A. Cama, R. M. Chesnut, P. De Bonis, A. Estraneo, A. Figaji, S. I. Florian, R. Formisano, P. Frassanito, C. Gatos, A. Germanò, C. Giussani, I. Hossain, P. Kasprzak, F. La Porta, D. Lindner, A. I. R. Maas, W. Paiva, P. Palma, K. B. Park, P. Peretta, A. Pompucci, J. Posti, S. K. Sengupta, A. Sinha, V. Sinha, R. Stefini, G. Talamonti, A. Tasiou, G. Zona, M. Zucchelli, P. J. Hutchinson, F. Servadei

**Affiliations:** 1 Division of Neurosurgery, Department of Biomedical, Metabolic and Neural Sciences, University Hospital of Modena, University of Modena and Reggio Emilia, Modena, Italy; 2 Division of Neurosurgery, Department of Clinical Neurosciences, Addenbrooke’s Hospital, Cambridge Biomedical Campus, University of Cambridge, Box 167, Cambridge, CB2 0QQ UK; 3 NIHR Global Health Research Group on Neurotrauma, University of Cambridge, Cambridge, UK; 4 Division of Neurosurgery/Children’s Neuroscience, Department of Child Health, Barrow Neurological Institute, Phoenix Children’s Hospital, Phoenix, AZ USA; 5 Neurosciences Institute, Neurosurgery Service, MEDITECH-INUB Research Group, El Bosque Clinic, El Bosque University, Bogotá, Colombia; 6 Department of Neurosurgery, Pecs University, Pecs, Hungary; 7 Department of Neurosurgey, “Santobono-Posillipon” Children’s Hospital, Naples, Italy; 8 Department of Neurosurgery, Universityof Thessaly, University Hospital of Larissa, Larissa, Greece; 9 Northwest General Hospital and Medical Center, Peshawar, Pakistan; 10 Department of Neurosurgery, “Sant’Eugenio” Hospital – ASL ROMA 2, Rome, Italy; 11 Neurosurgery Division, Faculty of Medicine, University of Malaya, Kuala Lumpur, Malaysia; 12 Department of Neurological Surgery, University College Hospital, College of Medicine, University of Ibadan, Ibadan, Nigeria; 13 Division of Neurosurgery, University of São Paulo Medical School, São Paulo, SP Brazil; 14 Neurosurgical Unit, “SS. Antonio e Biagio e Cesare Arrigo” Hospital, Alessandria, Italy; 15 Department of Pediatric Neurosurgery, Institute for Scientifict Research and Care “Giannina Gaslini”, Genua, Italy; 16 Department of Neurological Surgery, School of Global Health Harborview Medical Center, University of Washington, Seattle, WA USA; 17 Department of Orthopaedic Surgery, School of Global Health Harborview Medical Center, University of Washington, Seattle, WA USA; 18 Department of Neurosurgery, Sant’Anna University Hospital, Ferrara, Italy; 19 IRCCS Fondazione Don Carlo Gnocchi, Florence, Italy; 20 Neurology Unit, Santa Maria della Pietà General Hospital, Nola, Italy; 21 Division of Neurosurgery, Neuroscience Institute, University of Cape Town, Red Cross Children’s Hospital, Rondebosch, Cape Town, South Africa; 22 Department of Neurosurgery, Iuliu Hatieganu University of Medicine and Pharmacy, Cluj Napoca, Romania; 23 Post-Coma Unit, IRCCS Santa Lucia Foundation, Rome, Italy; 24 Department of Pediatric Neurosurgery, “University Cattolica del Sacro Cuore- School of Medicine “A. Gemelli”, Rome, Italy; 25 Department of Neurosurgery, University Hospital of Thessaly, University Hospital of Larissa, Thessaly, Greece; 26 Neurosurgical Clinic, Department of Neurosciences, University of Messina, Messina, Italy; 27 Neurosurgery, Department of Surgery and Translational Medicine, Milan Centre for Neuroscience, University of Milano-Bicocca, San Gerardo Hospital, Monza, Italy; 28 Department of Neurosurgery, Division of Clinical Neurosciences, University of Turku and Turku University Hospital, Turku, Finland; 29 Department of Neurosurgery, Medical University of Lodz, Łódź, Poland; 30 Neurorehabilitation Unit, IRCCS Istituto delle Scienze Neurologiche di Bologna, Bologna, Italy; 31 Department of Neurosurgery, University Hospital Leipzig, Leipzig, Germany; 32 Department of Neurosurgery, Antwerp University Hospital and University of Antwerp, Edegem, Belgium; 33 Division of Neurosurgery, Hospital das Clínicas, Faculdade de Medicina da Universidade de São Paulo, São Paulo, Brazil; 34 Department of Pediatric Neurosurgery, “Bambin Gesù” Children’s Hospital, Rome, Italy; 35 Program for Global Surgery and Social Change, Harvard Medical School, Boston, MA USA; 36 Department of Pediatric Neurosurgery, “Città della Salute e della Scienza di Torino - Presidio ospedaliero Regina Margherita”, Turin, Italy; 37 Department of Neurosurgery, ASL Latina “Santa Maria Goretti” Hospital, Latina, Italy; 38 Department of Neurosurgery and Turku Brain Injury Centre, Turku University Hospital and University of Turku, Turku, Finland; 39 Department of Neurosurgery, Command Hospital Kolkata, Kolkata, India; 40 Department of Neurosurgery, The Walton Centre NHS Foundation Trust, Liverpool, UK; 41 Department of Neurosurgery, SMS Medical College and Hospital, Jaipur, Rajasthan India; 42 Departiment of Neurosurgery, ASS T-Ovest Milanese, Legnano Hospital, Milan, Italy; 43 Department of Neurosurgery, “Niguarda” Hospital, Milan, Italy; 44 Division of Neurosurgery, Department of Neurosciences (DINOGMI), IRCCS Ospedale Policlinico San Martino, Genoa, Italy; 45 Dipartimento di Neuroscienze, Riabilitazione, Oftalmologia, Genetica e Scienze Materno Infantili (DINOGMI), IRCCS Ospedale Policlinico San Martino, Università di Genova, Genoa, Italy; 46 Section of Pediatric Neurosurgery, Institute for Scientifict Research and Care “Bellaria”, Bologna, Italy; 47 Humanitas Clinical and Research Center-IRCCS, Rozzano, Milan, Italy; 48 Department of Biomedical Sciences, Humanitas University, Rozzano, Milan, Italy

**Keywords:** Cranioplasty, Rehabilitation, hydrocephalus, Decompressive craniectomy, Neurosurgery

## Abstract

**Background:**

Due to the lack of high-quality evidence which has hindered the development of evidence-based guidelines, there is a need to provide general guidance on cranioplasty (CP) following traumatic brain injury (TBI), as well as identify areas of ongoing uncertainty via a consensus-based approach.

**Methods:**

The international consensus meeting on post-traumatic CP was held during the International Conference on Recent Advances in Neurotraumatology (ICRAN), in Naples, Italy, in June 2018. This meeting was endorsed by the Neurotrauma Committee of the World Federation of Neurosurgical Societies (WFNS), the NIHR Global Health Research Group on Neurotrauma, and several other neurotrauma organizations. Discussions and voting were organized around 5 pre-specified themes: (1) indications and technique, (2) materials, (3) timing, (4) hydrocephalus, and (5) paediatric CP.

**Results:**

The participants discussed published evidence on each topic and proposed consensus statements, which were subject to ratification using anonymous real-time voting. Statements required an agreement threshold of more than 70% for inclusion in the final recommendations.

**Conclusions:**

This document is the first set of practical consensus-based clinical recommendations on post-traumatic CP, focusing on timing, materials, complications, and surgical procedures. Future research directions are also presented.

## Introduction

Decompressive craniectomy (DC) is a surgical procedure frequently used following traumatic brain injury (TBI) [6, 100]. Decompressive craniectomy can control intracranial pressure (ICP) and reduce mortality with a wide range of outcome categories in survivors, but the related complications are not insignificant [29, 36]. While there has been a significant amount of literature devoted to DC for TBI, the available guidelines [6] do not include recommendations on cranioplasty (CP), which is an operation aiming to reconstruct the skull defect created by the DC.

As a consequence, several issues remain open to debate, including indications, optimal surgical technique, optimal material, timing of CP, CP with concomitant hydrocephalus (HC), and CP in paediatric patients [13, 18, 23, 37, 47, 69, 75, 93, 94].

When health issues are particularly complex or controversial, and high-quality evidence to inform evidence-based guidelines is scarce, a practical and effective solution can be a consensus-based process. The latter is an established and formal methodological tool for reaching consensus among professionals on the basis of the best available evidence or practice. Typically, a panel of experts, after examining the relevant scientific information and the clinical issues, produce clinical recommendations which reflect the commonly held views, in order to guide practice in relationship to the available resources [39].

The main objective of the international consensus meeting on cranioplasty was to develop recommendations by using a consensus-based approach in order to optimize the management of TBI patients requiring CP*.*

## Methods

### Supporting agencies

The consensus meeting was held in Naples, Italy, on June 21–22, 2018. The event was organized during the International Conference on Recent Advance in Neurotrauma (ICRAN), the conference of the Neurotrauma Committee (NTC) of the World Federation of Neurosurgical Societies (WFNS). ICRAN 2018 was held under the patronage of the Italian Neurosurgical Society (SINch), the European Association of Neurological Surgeons (EANS), the International Neurotrauma Society (INTS), GLOBAL NEURO, the Italian Society of Anaesthesia Analgesia Resuscitation and Intensive Care (SIAARTI), the Italian Society of Neurological Rehabilitation (SIRN), the Italian Agency for Development Cooperation (AICS), and the National Institute for Health Research Global Health Research Group on Neurotrauma (NIHR GHRGN). The NTC of WFNS and the NIHR GHRGN supported the consensus meeting.

### Preparatory work

In advance of the meeting, the organizing committee developed and agreed the 5 fundamental topics that were believed to require consideration: (1) indications and technique of CP, (2) materials, (3) timing, (4) post-traumatic HC in patients following DC, and (5) CP in paediatric patients.

### Consensus meeting

The consensus meeting started with an open session of oral presentations, as part of in the ICRAN 2018 programme, with an update on the recent literature on the topics of DC following TBI and subsequent CP.

### Participating experts and working groups

The consensus meeting participants were invited with the aim of achieving broad geographic representation. They were divided into five small working groups according to the five pre-specified topics. Each working group met in a separate room with the aim of discussing available published evidence as well as their personal clinical experience. Then, under the coordination of two facilitators, each small group summarized in a document the identified areas of agreement/disagreement, and a preliminary list of draft recommendations for clinical practice and areas requiring future investigation. Besides the international panel of neurosurgical experts, Physical Medicine and Rehabilitation (PM&R) Physicians were involved as C3 participants and invited in the small groups discussing HC and timing of CP.

### Grading of approval methods

Following small group discussions, all invited delegates participated in the next session, where the facilitators presented the summary documents of their small group. The statements were voted in real-time and anonymously by all invited participants using an audience engagement software (Glisser Limited, London, UK). The appendix (available online) includes a summary of all accepted statements and statements that reached a poor level of consensus.

The degree of agreement was expressed with a three-point Likert scale (agree, uncertain, and disagree). A minimum of 70% agreement for each statement was required for inclusion in the final consensus statement. Recommendations which during the first round of voting had levels of agreement close to 70% (i.e. just above or just below), high levels of uncertain responses, or similar levels of agreement and disagreement were refined following floor discussion and put to a second round of voting. A similar methodology has been previously adopted and reported by the International Consensus Meeting on the Role of Decompressive Craniectomy in the Management of TBI held in Cambridge in September 2017 [35].

The decision to adopt a consensus-based methodology as opposed to an approach such as GRADE, for example, was based on the fact that in the field of cranioplasty the vast majority of studies are retrospective observational. Only 2 pilot randomized trials have been published so far and the overall level of evidence remains low.

## Consensus topic 1: Indications and technique

### Overview

The term post-traumatic CP refers to reconstruction of the cranial vault following DC for TBI, which takes place at a later stage. Craniectomies can be performed early or late, as primary or secondary interventions [52]. As there is a general hiatus in the literature to define the actual size and/or extent (i.e. uni/bilateral), when post-traumatic DC is performed, the actual size of reported CPs varies widely [75]. However, it important to remark that either small or large cranial defects may be related to significant neurological and psychological consequences [21, 27]. A clear-cut definition regarding size has been recommended by the Guidelines for the Management of Adult Severe TBI (4th Edition) for frontotemporoparietal DC (not less than 12 cm × 15 cm, or 15 cm diameter) over a small frontotemporoparietal DC to reduce mortality and improve neurologic outcomes in patients with severe TBI [6].

Recent trials have led to an increase in the use of DC in the acute management of severe TBI and other pathologies, even in areas of limited surgical resources [11]. The expanding number of DCs has been coupled with inconsistencies in operative techniques. A fundamental key point to keep in mind is that since DC requires a second surgery for CP, the decision making for CP should begin at the time of the actual decompression. This would entail surgical planning based on the original skin incision taking into account potential scalp trauma, size, and location of the DC to manage the acute pathology, type, and need for dural and scalp closure and timely identification and treatment of the acute complications of the DC. All these features will ultimately define the potential success of CP.

The broadly accepted indications for CP include the following: reconstruction of the cranial defect, prevention or treatment DC-related complications including extracerebral fluid collections, altered cerebrospinal fluid (CSF) flow, and/or absorption resulting in HC, affected self-esteem/psychological consequences, barotrauma-related inhibition of rehabilitation, and “sinking skin flap syndrome” [46].

### Voting results

1. The discussion is focused on post-traumatic cranioplasty (CP) after DC. [Agree 97.2%]
2. The discussion covers cranial defects following bifrontal or fronto-temporo-parietal decompressive craniectomy (DC). [Agree 97.4%]
3. Every effort should be made to perform a CP after DC in the absence of medical contraindications and following consent. [Agree 100%]
4. Indications include anatomical reconstruction (CSF barrier, bone, and soft tissue), physiological restoration, and to promote functional and psychological recovery. [Agree 100%]
5. Use of 3D planning techniques, when available, may aid the surgical procedure. [Agree 86.5%]
6. Some fullness of the flap is not per se a contraindication to CP. [Agree 81.6%]
7. Perioperative (temporary) CSF diversion may be considered to aid CP. [Agree 92.3%]
8. Artificial implants and bone flaps with a gap to the surrounding skull should be secured. [Agree 87.2%]

### Future research directions

Heterogeneity of care is particularly prevalent in TBI [61]. In particular, inequality between various countries is reflected in the availability of resources (e.g. 3D planning/printing, PEEK/titanium implants). While high-end techniques are available in a small fraction of societies, the majority of DC takes place in low- and middle-income countries where the incidence of TBI is the highest and DC is often used as the primary way of treating severe TBI. Cost efficiency is a real issue and cheaper solutions may not necessarily stand up to long-term scrutiny. While titanium implants are more expensive than autologous bone, the latter might be associated with higher rate of infection thereby leading to a cost-benefit equipoise [99].

Potential future research directions, especially for resource-limited settings, should be developed for alternative techniques to avoid or reduce additional costs (e.g. bone flap storage or alloplastic prosthesis) and second surgeries as widely discussed in literature (e.g. hinge CP and variants such as four quadrant or expansive CP) [2, 48, 80, 85].

## Consensus topic 2: Materials

### Overview

Despite a long-lasting debate, no agreement has been reported in the literature regarding what is the best cranioplasty material [86]. The majority of debates in literature concern the optimal CP material and revolve around the choice between autologous bone and other biomaterials, either biologic or synthetic ones [31]. This area is wide open to further development and refinement when it comes to CP procedures.

#### Autologous bone

Autologous bone has been the most commonly used material for reconstruction CP due to the perceived higher grade of biocompatibility and its low cost [22, 28, 47, 68]. Nevertheless, autologous bone is the only CP material associated with a specific material-related complication: bone flap resorption (BFR) [89]. This is highly relevant in terms of outcome due to the possible need for further surgery, with reoperation and replacement using an alloplastic material, especially if there is structural breakdown due to BFR. The aetiology for BFR can be variable and likely include infection, lack of sufficient scalp and or dural blood supply, and lack of incorporation to the surrounding bone.

Following bone removal, storage of the bone flap either in a subcutaneous or submuscular abdominal pocket or in extra-corporeal situations bone biobanking is required. The latter choice in some countries is mandatory due to national rules, and it has been thought to be the main cause of BFR. Nevertheless, in a systematic review Corliss et al. found no statistically significant differences in terms of infection, resorption, and/or reoperation rate comparing extra-corporal cryopreservation versus abdominal pocket storage [13].

BFR though remains a significant concern when using autologous bone. In a recent systemic review and meta-analysis [62], an increased risk of reoperation for autologous implant mainly related to BFR was reported, with no increased infection rates compared with synthetic material. Younger age, shunt dependency, and bone flap fragmentation as independent risk factors for BFR were also reported [53, 81]. A prospective multi-centre study showed the development of major complications mainly due to BFR [38].

#### Polymethylmethacrylate

Polymethylmethacrylate (PMMA) is a non-absorbable, radiolucent, inert, and very common alloplastic material for CP [22, 47]. Liquid PMMA has the advantages of being easily moldable and inexpensive, providing an attractive solution for those cases in resource-poor regions. For patients who have failed multiple previous attempts at CP because of surgical site infection (SSI), the liquid PMMA can be impregnated with antibiotics and could represent an additional CP option [32].

The 3D-solid customized PMMA prosthesis [24] eliminated the disadvantage of liquid PMMA, such as the exothermic reaction during polymerization, the surgeon dependency of the intra-operative preparation, the relative contraindication in pregnancy, and the fact that toxic fumes are released.

#### Titanium

Titanium CP can be utilized for CP as a plate, mesh, or 3D porous implant. Hardness and stability are the main advantages of this material [33]. Titanium plates constitute the most common solution, with better cosmetic and functional outcomes, compared to autologous bone without increasing overall health care costs as reported in a recent randomized controlled trial [31]. However, non-physiological heat conduction, radiopacity with significant artefact, and difficulty intra-operatively shaping are the main disadvantages. Titanium meshes are more heterogeneous in terms of thickness, stiffness, and degree of conformability. They can be manually shaped, taking into account that the wider the defect, the less hard and more malleable is the mesh. Preoperative radiotherapy and free flap coverage were associated with complications, and an increased incidence of soft tissue atrophy was associated with the use of titanium mesh in a large series of 50 patients, although the titanium mesh was generally well tolerated [10].

Most recently 3D porous titanium has been suggested as a reliable alternative [19]. Despite its high cost and limited literature available, 3D porous titanium shows promising results about the achievement of most complex skull reconstructions with a shorter operative time.

#### Polyetheretherketone

Polyetheretherketone (PEEK) is a more recent material that is also presently utilized for CP. It is also an inert, durable, and mechanically sound material, but at the time of this consensus conference, there were no studies on its long-term efficacy and complication rate. A trend toward decreased post-operative complication rates as compared to autologous grafts has been reported in a systematic review and meta-analysis [77]. Additionally, the rate of overall complications including post-operative new-onset seizures, postoperative implant exposure, and reoperation after CP was reportedly lower in a PEEK CP group than in those patients treated with titanium CP [102]. Required in-house sterilization and a possibility of increased seroma formation due to the smooth surface could represent the main disadvantage of this material.

#### Porous hydroxyapatite

Hydroxyapatite (HA), makes up the main component of the substitute bone implant. Approximately 50% of the dry weight of bone matrix is inorganic materials, the most abundant of which is HA [41]. An advantage of HA is its biocompatibility due to the absence of host immune interactions or systemic/local toxicity [9]. Osteoconductivity and biocompatibility of porous HA implants have been confirmed in multiple studies [72].

Fracture of the prosthesis in the first months after implant due to its porous structure is the main reported disadvantage of this material. Nevertheless, the specific property of porous structure provides the possibility of self-repair following head trauma and fracture [87]. A postoperative analysis of complications in 1549 patients showed a fracture incidence of 2.1%, and a re-operation incidence for fracture of 0.9% [88]. Among fractured prostheses, there was a re-operation incidence of 44.4% as well as a self-repaired occurrence of 18.1% observed. A pilot randomized trial of HA vs titanium found that the rate of local implant-associated wound infection in the HA group was 2 of 26 (7.7%) patients and 5 of 24 (20.8%) patients in the titanium group (*p* = 0.407). In both groups, 7 patients required reoperation at the 6-month follow-up (26.9% of the HA group and 29.2% of the titanium group). Due to the limited sample size, these results need to be interpreted with caution [60].

### Complications related to materials for CP following post-trauma DC

Complications following CP can result from the intervention itself, or as a result of factors apparently unrelated to the CP procedure. Thus, separating complications related to CP from those deriving from the clinical history of the patient from the initial bone removal remains a challenge, and, as evidenced by our discussions, at present there is no clear superiority of one material over others.

Predictors of CP complications are as follows: previous reoperation, comorbidities such as unhealthy body mass index (BMI), smoking, diabetes in the case of stroke patients as well the presence of a ventriculoperitoneal shunt (VPS), as reported by Walcott et al. [96]. Skin flap complications following DC and CP have also been reported [18], but still no relationship has been reported among the different biomaterials used for the CP and type and extent of complications.

The location of the DC appears related to CP complications. Analyses of series using varying biomaterials found that bifrontal CP was associated with a 2-fold increased risk of complication and a 2.5-fold increased risk of developing infection compared with hemispheric/bihemispheric CP [16], using different CP materials. A retrospective review of autologous CP reported that 67% of 12 bifrontal compared to 16% of 49 unilateral autologous CP required reoperation (*p* < 0.01) [23].

In a large series of 98 PMMA CP and minimum 2-year follow-up, there were 9 removed implants, 8 as a result of infection due to direct frontal sinus involvement [9]. The largest incidence of infection was in patients with a bifrontal defect (3.8%) in an analysis of 1549 patients with HA implants [88]. A large retrospective UK study on 174 titanium CP to date [69] showed that bifrontal insertion is one of the most relevant risk factors for complications with a complication rate of 40%.

Bone or alloplastic implant removal until complete healing of the surgical field has occurred is the usual management of an infected CP. In a close future, a project to develop materials capable of resisting colonization and more effective pharmacological treatment against infections will be necessary.

In a recent study of four cases of infectious complications, it has been suggested that porous HA CP may be utilized for improved prosthesis retention management [37] due to a lower biofilm colonization rate and increased revascularization. It was suggested that the revascularization resulted in improved “in situ” intake of antibiotics due to the porous structure, and in combination with targeted antibiotic therapy, may prevent the need for prosthesis removal.

This concept might lead to some promising future applications using porous surface material such as HA and 3D porous titanium CPs.

### Voting results

1. The question of the optimal material for cranioplasty (i.e. bone graft, synthetic) with regard to clinical outcome, cosmesis, and complications remains unanswered. [Agree 88.5%]
2. Bone graft carries the risk of resorption. [Agree 100%]
3. The influence of storage methods on resorption rates is unclear. [Agree 88.5%]
4. Progressive resorption over time can affect bone stored subcutaneously, so early cranioplasty can be considered. [Agree 96%]
5. The influence of timing of cranioplasty on resorption rates of bone graft remains unclear. [Agree 88.5%]
6. Custom-made implants may have better cosmetic outcomes and this should be studied further. [Agree 92%]

### Future research directions

The application of nanotechnologies to HA/polymer composite will undoubtedly be key to optimizing HA as a biocompatible material of choice [25]. Even if porous titanium by itself is inert and shows poor surface activity without surface biomodification, some bone in-growth rate (BIR) is still observed without surface biomodification. However, a substantial BIR is seen when HA is Si-HA coated, the SI-HA-coated porous titanium showing the highest BIR [101]. Almost all materials are now being deployed to produce 3D CP patient specific implants (PSI). It has been suggested that this will improve cosmetic benefit, but it has not yet been proven to have a positive impact on outcome. 3D-printing and stereolithography application in neurosurgery [3] could have a growing use, such as to create moulds to be filled with liquid PMMA [7, 58], to obtain an intraoperative symmetric CP not limited by the manual skill of the surgeon. Titanium meshes may be stereo-lithographically created using direct metal laser sintering in order to create a structural foundation [10, 65], while custom-made porous titanium graft can be shaped with electron beam melting technology [19].

In the near future, the largest barriers to artificial implants such as the costs, low confidence as to improved outcomes by clinicians, and government and health insurance for printing [74] will hopefully be reduced with increased widespread adoption of this technology.

Further evidence will be required with regard to the optimal material for cranial reconstruction.

## Consensus topic 3: Timing

### Overview

Literature meta-analysis, reported retrospective cohorts, and prospective studies did not define a clear correlation among time interval between bone removal and CP and any resultant major complications and outcome [14, 16, 55, 68, 76]. In the literature, the definition of “early CP” is ill-defined and has been reported at various time intervals varying from less than 4 weeks up to 12 weeks. Because of this lack of consistency in the literature, the impact of timing on functional outcome is not well defined from literature data [22, 31, 47, 86]. However, a recent comprehensive systematic review concluded that CP may “improve neurological function, and earlier cranioplasty may enhance this effect” [63].

A lower rate of complications [8, 78, 79, 82, 98] associated to early CP has been described. However, some authors [83, 91] have found that early CP is associated with more complications, in particular the higher rate of infection between 0 and 6 months.

A correlation between good outcome and benefits from CP such as improved postural blood flow, cerebrovascular reserve capacity, and cerebral glucose metabolism is far from having been established but has been preliminarily suggested. It is therefore just logical to consider that the earlier the CP, the sooner it is performed; there may be additional benefits to recovery by this yet to be proven [40, 59].

The ideal timing among several studies would seem to suggest that the time-window is probably not the same for each patient, and may be related to the individual patient’s clinical condition including the presence of haemodynamic or respiratory instability, infection status, a clinical diagnosis of minimally conscious or vegetative states [20, 34, 92], delayed wound healing from the initial surgeries or other trauma-related surgeries (i.e.) abdominal, chest, orthopaedics, etc., ongoing bleeding diathesis, or any condition related to the trauma itself such as the brain condition at the moment of proposed CP.

The timing of CP should also take into account the state of the skin flap: (i) depressed skin flap, due to post-traumatic brain atrophy or over-drainage of CSF related to VP shunting; (ii) skin flap is at the same level as the margins of the cranial vault; (iii) skin flap is bulging beyond the cranial vault margin due to brain swelling and/or HC/ventriculomegaly (VM).

### Voting results

1. Cranioplasty may improve neurological function, and earlier cranioplasty may enhance this effect. [Agree 84.6%]
2. Defining a threshold for early vs late cranioplasty is artificial but can be useful for clinical benchmarking and research purposes. Taking into account the previous statement, ultra-early cranioplasty is up to 6 weeks following craniectomy, early cranioplasty is 6 weeks to 3 months, intermediate 3–6 months, and delayed more than 6 months. [Agree 86.8%]
3. The clinical condition of the patient (e.g. wound status, systemic infection, systemic instability, antithrombotic medications) should be taken into consideration when deciding the timing of cranioplasty. [Agree 100%]
4. Poor neurological status is not a contraindication for cranioplasty per se. [Agree 92.1%]
5. Consider expediting the cranioplasty in a patient with neurological and/or neuropsychological deterioration that cannot be attributed to extracranial causes. [Agree 81.6%]
6. The odds of infections, reoperations, intracranial haemorrhage, and seizures do not appear different between early and late cranioplasty. [Agree 88%]
7. Progressive resorption over time can affect bone stored subcutaneously, so early cranioplasty can be considered. [Agree 96%]
8. Skin colonization (i.e. carrier) of the patient is not a contraindication for cranioplasty but consideration to decolonization pre-operatively should be given. [Agree 92%]
9. The effect of timing on neurological outcome should be explored in future, prospective studies. [Agree 89.7%]
10. The relationship between the timing of CP and HC needs to be explored in future studies. [Agree 89.5%]
11. The development of specific outcome measures following cranioplasty should be considered. [Agree 94.7%]

### Future research directions

At present, there is an ongoing prospective, multi-centre, observational study on outcomes CP (UK Cranioplasty Study) that may be able to shed some light on the issue of timing [50].

Future studies evaluating the relationship between the timing of CP on neurological outcome and or the development of HC are necessary, especially randomized trials.

## Consensus topic 4: Hydrocephalus

### Overview

The development of ventriculomegaly (VM) after DC for severe TBI is well documented. Its incidence significantly varies among the reported series from 40 to 45% [30, 64]. However, among the patients that develop VM following TBI, only a small percentage require the insertion of a ventriculo-peritoneal (or less frequently lumbar-peritoneal) shunt for its management [30, 64, 95]. This discrepancy requires to differentiate the features of clinically proven hydrocephalus (HC), which requires surgical treatment, to those of VM, which refers to ex vacuo dilatation of the ventricular system resulting from brain atrophy due to severe TBI.

Even after differentiating HC from VM, the actual incidence of HC among patients underwent DC for TBI varies significantly among the published series (10–45%) [30, 43, 70, 95]. The vast majority of the published series are retrospective studies, carrying all the weaknesses and the potential biases related to their retrospective nature. There are only two retrospective studies with prospectively collected data in the pertinent literature [93, 95]. Moreover, the number of different criteria for diagnosis of VM or HC following post-traumatic DC may represent another factor to explain the wide range in the rate of incidence of HC [12, 17, 30, 90].

Several predisposing factors for the development of HC following posttraumatic DC are summarized in Table 1.

**Table 1:** Predisposing factors for post-DC hydrocephalus

Interhemispheric hygroma development	
Subdural hygroma development	
Low GCS score upon admission*	
Increased ICP before DC	
Elderly patients	
Proximity of the DC (< 2.5 cm) to the anatomical midline	
Delayed (> 3 months) CP	

*According to classification of TBI severity based on GCS

Several reported series have demonstrated an association between post-DC HC and factors including the development of an interhemispheric hygroma [17, 42, 70, 95], or subdural hygroma [30], severity of the initial injury as reflected by a low admission GCS score and the increased ICP during the acute course [1, 42]. Higher risk of developing post-DC HC for younger patients [70, 95], and for bifrontal DC in comparison to unilateral DC has been reported, although not statistically significant [30]. A statistically significant association between distance of the DC border less than 2.5 cm to the anatomical midline and the development of HC has been reported [17].

The time interval between the DC and the development of HC varies between the reported series, with a median time interval of 6.4 months (range 1–15) or 43.7 days (range 23.5–199), but it seems that HC takes at least 1 month to develop after DC [70, 95]. Interestingly, the previously published series agreed on the finding that the vast majority of patients (> 80%) who develop an interhemispheric hygroma after a DC will develop HC within 50 days from the DC [17, 42, 70, 95].

The development of specific diagnostic criteria for HC in the context of post-decompression VM should be further studied. The establishment of the diagnosis of post-DC HC requires both the presence of clinical symptoms and signs, along with radiologically proven VM [70].

However, it is unlikely that the post-DC HC will present with the typical clinical triad observed for the idiopathic normal pressure hydrocephalus (cognitive deterioration, gait abnormalities, and urinary incontinence), as the underlying TBI is likely to have caused severe neurological impairments that submerge this typical symptomatology [97]. Thus, HC should be suspected, in the presence of VM, when patients fail to have the expected recovery following the initial event (exceedingly slow or arrested recovery) or when preexisting cognitive and/or motor deficits begin to deteriorate (progressive neurological deterioration) or even in case of atypical symptoms of new onset such as seizures [15].

The onset of the aspecific HC symptomatology often takes place during the post-acute phase, where patients with DC are often admitted to specialized intensive rehabilitation, in view of the complex disabilities following TBI [45, 54, 66]. As the clinical presentation of HC is often subtle, there is a need to document the arrested recovery or the progressive neurological deterioration caused by HC using clinical scales. These scales should be able to assess the patient’s functional status over time and that are enough responsive to the subtle and mild changes of the patient’s neurological condition. For instance, the Coma Recovery Scale-Revised (CRS-R) is definitely appropriate to detect even subtle neurological changes in patients with post-traumatic DC affected by a disorder of consciousness (DOC) [84]. However, the same scale is not appropriate for patients who have emerged from DOC being in a post-traumatic confusional state. Indeed, clinical scales which are targeted to the functional level of the patients should be used. In this respect, an outcome scale such as the Glasgow Outcome Scale, even in its extended form, is unlikely to be enough sensitive to change to capture these subtle changes and, thus, its use cannot be recommended. For these reasons, the operationalization of the diagnostic criteria for HC in the context of post-decompression VM should be further studied.

However, early recognition of post-DC HC is of paramount importance, since its proper management can improve the patient’s neurological status and her/his overall outcome [54].

All previous reports and reviews agreed on the fact that the development of post-DC HC is associated with worse outcome [17, 30, 42, 70]. Previous series have shown that delayed restorative CP (more than 3 months after the DC) is associated with HC [70]. However, other series concluded that timing of CP was not associated with the development of HC [1, 30, 42]. A more recent systematic review concluded that CP within 90 days after DC in TBI patients alone was associated with a lower incidence of hydrocephalus [71].

The disappearance of VM after CP is well documented. Thus, the existing controversies about the proper management for HC and CP regard the timing of CSF diversion with respect to the cranial reconstruction. When a CSF shunt is required, no evidence exists in the literature regarding the best time option (before, synchronously, or after CP) for insertion [44, 51, 56, 57, 60, 70, 73, 93].

A CP before the VPS may prolong and intensify the effect of HC to the brain [98], but at the same time may increase the technical difficulty of a subsequent CP [59]. In a previously published study, a higher incidence of anaesthesia-related and prophylactic antibiotic–related complications is reported [40]. A CP performed after VPS has been reported to be associated with higher complication rate, and an increased shunt revision rate [59]. In cases of simultaneous performance of VPS and CP, higher perioperative complications, including higher infection rates, have been reported [26, 40], although other reports found the incidence to be similar [67].

Based on a number of case series, it has been suggested that programmable shunts could be effective in the management of HC following post-traumatic DC. Nevertheless, a fixed pressure-valve is a well-recognized treatment in socio-economic environments with limited resources. This is especially significant given the availability of a number of lower-cost fixed-pressure shunts that may be more useful in the setting of lower resources [40, 59].

### Voting results

1. With regard to the best imaging method for following patients with VM, there is not adequate evidence in the literature. [Agree 81.6%]
2. The performance of serial cranial imaging (e.g. CT, MRI) can provide information regarding any changes in the patient’s ventricular system. [Agree 81.6%]
3. With regard to the best electrophysiological test for following patients with ventriculomegaly, there is not adequate evidence in the literature identifying such a modality. [Agree 92.1%]
4. The optimal management of decompressed patients with ventriculomegaly remains uncertain, but the performance of a cranioplasty prior to definitive CSF diversion should be considered as it can help restore an intact intracranial system. [Agree 95.8%]
5. In those patients that there is a bulging skin flap, the usage of a temporary external ventricular drain (EVD) or a lumbar drain (LD) may facilitate the cranioplasty. The performance of serial spinal taps, although is not supported by enough evidence, is an option to facilitate the cranioplasty. [Agree 94.7%]
6. After the performance of cranioplasty, the patient should be closely followed for signs of HC. In case of persisting or developing HC, CSF diversion needs to be considered. [Agree 92.1%]
7. The development of specific diagnostic criteria and outcome measures for post-traumatic HC should be a high priority. [Agree 84%]
8. The development of specific diagnostic criteria for HC in the context of post-decompression VM should be studied further. [Agree 100%]
9. CSF infusion studies may be helpful in patients with VM to determine the presence of HC, after a cranioplasty has been performed. [Agree 95%]

### Future research directions

Clinical studies of high methodological quality evaluating the relationship between CSF disorders after DC and the impact of CP are required. Studies looking for the development of specific diagnostic criteria and outcome measures for post-traumatic HC should be a high priority in order to use these criteria in subsequent research.

## Consensus topic 5: Paediatric

### Overview

TBI is the most common cause of death and disability in children. The most recent edition of the paediatric guidelines for the management of severe TBI has recommended the use of DC in order to reduce intracranial hypertension as a second tier therapy [43, 49].

Following resolution of the acute period of the secondary injury phase associated with intracranial hypertension, there needs to be consideration for reconstruction of the surgical defect [4]. Post-traumatic CP as noted above is a routine neurosurgical procedure to restore CSF dynamics and cosmesis, and to provide cerebral protection, all which can facilitate improved neurologic rehabilitation as well as an improved neurologic outcome, particularly in the paediatric population.

As paediatric post-traumatic CP is not as common an occurrence as in the adult, it is necessary to provide general guidance for all controversies as well as the role of specialty paediatric centers for definitive care. The literature to date has not helped to define a clear optimal strategy; specifically [5, 20] , there has been no consensus reported about the type of surgical approach for the dissection/reconstruction of the defect, the type of materials, and the optimal timing when the CP procedure should be performed.

### Voting results

#### Defining the parameters and indication of paediatric post-traumatic paediatric ICP

1. The paediatric population for the purposes of this consensus statement is defined as children less than 18 years of age. [Agree 78.9%]
2. All statements could be applied to bifrontal and fronto-temporo-parietal cranioplasties. [Agree 94.7%]
3. The assumption is that the decompressive craniectomies for children especially young children were proportional to size of the child. The cranioplasty/reconstruction would be proportional to the size of the defect. [Agree 100%]
4. Following DC, there should be no age limit for reconstruction. [Agree 92.1%]

#### Materials in a post-traumatic paediatric CP

1. After DC, autologous bone is preferred for all ages of children. [Agree 78.4%]
2. If autologous bone is not available, an osteoconductive material should be preferred for reconstruction. [Agree 84.2%]
3. Below 3 years of age, the best option for osteoconductive material remains unclear. [Agree 84.2%]
4. If a child is more than 3 years of age, and an osteoconductive material is not available, a synthetic material can be used but the best option for synthetic material remains unclear. [Agree 89.5%].

#### Timing of post-traumatic paediatric CP

1. Systemic, neurologic, and wound conditions must be stable prior to consider cranioplasty. [Agree 81.6%]
2. There is uncertainty about the optimal timing for CP in paediatric population. [Agree 81.6%]
3. Recent neuroimaging is suggested prior to CP but there is uncertainty about the optimal neuroimaging modality (e.g. CT, MRI, ultrasound). [Agree 78.4%]

### Future research directions

Since there is limited literature in the area of paediatric CP, it was felt that there were a number of issues that could be addressed through future research. These include the surgical technique both for DC and the follow-up reconstructive CP, especially in children less than 1 year of age. Since the consensus was that the optimal materials for paediatric CP remains unclear, comparisons between autograft, allograft, osteoconductive materials, and synthetic materials would be useful in paediatric patients to determine whether there is indeed any difference in overall outcome, bony ingrowth, and complications both short-term and long-term.

It was also considered whether cadaveric allografts may be more beneficial in children because of their higher likelihood for bone incorporation though this too would require further research. Lastly since the optimal timing for CP in children remains unclear, a useful study could include the evaluation of outcome after the primary CP, comparing the different timing in reconstructive cases, those cases that have been complicated by severe cranial site infection requiring removal of the implant, and the best material for a secondary CP after these types of situations.

## Conclusion

This paper reports the final results of the first international consensus meeting on post-traumatic cranioplasty with the specific aim of developing recommendations based on literature reviews and experts’ opinions in order to facilitate the management of patients requiring CP globally.

Overall, 45 consensus statements were voted and agreed upon. Despite CP becoming a regularly common surgical procedure for patients undergoing DC following TBI, there are important gaps in the actual literature to define evidence-based recommendations for guiding clinical practice in different regions of the world. Hence, the objective of this process was to develop recommendations based on expert opinion in order to support decision making for day-to-day practice.

High-quality, methodologically robust, research is urgently required in many aspects of this procedure, such as alternative techniques of bone removal/cranial reconstruction, ideal material of cranioplasty, relationship between the timing of CP on neurological outcome, and or the development of HC with specific diagnostic criteria and outcome measures, in order to develop evidence-based recommendations and subsequent resource-stratified algorithms.

## Appendix

**Table 2:** Summary of all the statements voted during the consensus conference

**Indications and technique**	
• The discussion is focused on post-traumatic cranioplasty (CP) after DC. • The discussion covers cranial defects following bifrontal or fronto-temporo-parietal decompressive craniectomy (DC). • Every effort should be made to perform a CP after DC in the absence of medical contraindications and following consent. • Indications include anatomical reconstruction (CSF barrier, bone, and soft tissue), physiological restoration, and to promote functional and psychological recovery. • Use of 3D planning techniques, when available, may aid the surgical procedure. • Some fullness of the flap is not per se a contraindication to CP. • Perioperative (temporary) CSF diversion may be considered to aid CP. • Artificial implants and bone flaps with a gap to the surrounding skull should be secured.	
**Materials**	
• The question of the optimal material for cranioplasty (i.e. bone graft, synthetic etc) with regard to clinical outcome, cosmesis, and complications remains unanswered. • Bone graft carries the risk of resorption. • The influence of storage methods on resorption rates is unclear. • Progressive resorption over time can affect bone stored subcutaneously, so early cranioplasty can be considered. • The influence of timing of cranioplasty on resorption rates of bone graft remains unclear. • Custom-made implants may have better cosmetic outcomes and this should be studied further.	
**Timing**	
•Cranioplasty may improve neurological function, and earlier cranioplasty may enhance this effect. • Defining a threshold for early vs late cranioplasty is artificial but can be useful for clinical benchmarking and research purposes. Taking into account the previous statement, ultra-early cranioplasty is up to 6 weeks following craniectomy, early cranioplasty is 6 weeks to 3 months, intermediate 3-6 months, and delayed more than 6 months. • The clinical condition of the patient (e.g. wound status, systemic infection, systemic instability, antithrombotic medications) should be taken into consideration when deciding the timing of cranioplasty. • Poor neurological status is not a contraindication for cranioplasty per se. • Consider expediting the cranioplasty in a patient with neurological and/or neuropsychological deterioration that cannot be attributed to extracranial causes. • The odds of infections, reoperations, intracranial haemorrhage, and seizures do not appear different between early and late cranioplasty. • Progressive resorption over time can affect bone stored subcutaneously, so early cranioplasty can be considered. • Skin colonization (i.e. carrier) of the patient is not a contraindication for cranioplasty but consideration to decolonization pre-operatively should be given. [Agree92%] • The effect of timing on neurological outcome should be explored in future, prospective studies. • The relationship between the timing of CP and HC needs to be explored in future studies. • The development of specific outcome measures following cranioplasty should be considered.	
**Hydrocephalus**	
•With regard to the best imaging method for following patients with VM, there is not adequate evidence in the literature. • The performance of serial cranial imaging (e.g. CT, MRI) can provide information regarding any changes in the patient’s ventricular system. • With regard to the best electrophysiological test for following patients with ventriculomegaly, there is not adequate evidence in the literature identifying such a modality. • The optimal management of decompressed patients with ventriculomegaly remains uncertain but the performance of a cranioplasty prior to definitive CSF diversion should be considered as it can help restore an intact intracranial system. • In those patients that there is a bulging skin flap, the usage of a temporary external ventricular drain (EVD), or a lumbar drain (LD) may facilitate the cranioplasty. The performance of serial spinal taps, although is not supported by enough evidence, is an option to facilitate the cranioplasty. • After the performance of cranioplasty, the patient should be closely followed for signs of HC. In case of persisting or developing HC, CSF diversion needs to be considered. • The development of specific diagnostic criteria and outcome measures for post-traumatic HC should be a high priority. • The development of specific diagnostic criteria for HC in the context of post-decompression VM should be studied further. • CSF infusion studies may be helpful in patients with VM to determine the presence of HC, after a cranioplasty has been performed.	

**Table 3:** Summary of the statements concerning paediatric craniopasty

**Defining the parameters and indication of paediatric post-traumatic paediatric ICP** •The paediatric population for the purposes of this consensus statement is defined as children less than 18 years of age. • All statements could be applied to bifrontal and fronto-temporo-parietal cranioplasties. • The assumption is that the decompressive craniectomies for children especially young children were proportional to size of the child. The cranioplasty/ reconstruction would be proportional to the size of the defect. • Following DC, there should be no age limit for reconstruction.	
**Materials in a post-traumatic paediatric CP** •After DC, autologous bone is preferred for all ages of children. • If autologous bone is not available, an osteoconductive material should be preferred for reconstruction. • Below 3 years of age, the best option for osteoconductive material remains unclear. • If a child is more than 3 years of age, and an osteoconductive material is not available, a synthetic material can be used but the best option for synthetic material remains unclear.	
**Timing of post-traumatic paediatric CP** •Systemic, neurologic, and wound conditions must be stable prior to consider cranioplasty. • There is uncertainty about the optimal timing for CP in paediatric population. • Recent neuroimaging is suggested prior to CP but there is uncertainty about the optimal neuroimaging modality (e.g. CT, MRI, ultrasound).	

**Table 4:** Summary of statements that reached a poor level of consensus during voting sessions

**Indications and technique** •The panel did not reach a consensus on whether to include hinge CP in the statements.	
**Materials** •There is no evidence concerning storage methods and resorption rates. • There is no evidence of superiority of autologous bone compared to synthetic cranioplasties but autologous bone is associated with higher resorption rate. • Custom-made implants may have better cosmetic outcomes.	
**Timing** •Simple colonization without active infection is not a contraindication for cranioplasty. • There is no evidence of a difference in odds of infections, reoperations, intracranial haemorrhage, extra-axial fluid collections, and seizures between early and late cranioplasty. • A long delay between craniectomy and autologous cranioplasty (i.e. bone flap stored in the abdomen) is associated with a higher risk of bone resorption.	
**Hydrocephalus** • In regard to the best clinical test for following patients with ventriculomegaly, there is not adequate evidence in the literature identifying such a modality. The Glasgow Outcome Scale cannot detect mild changes in the patient’s neurological condition, even in its extended version. Therefore, its usage cannot be recommended. The employment of Coma Recovery Scale in patients with disorders of consciousness may detect even slight changes, and may assess over time the patient’s functional status.	
**Paediatric cranioplasty** •No statements	
